# Treatment Outcomes and Toxicities of Multiple Tyrosin Kinase Inhibitors for Metastatic Medullary Thyroid Cancer: A Case Series

**DOI:** 10.3390/biomedicines12122923

**Published:** 2024-12-23

**Authors:** Marilda Mormando, Rosa Lauretta, Giulia Puliani, Marta Bianchini, Maria Elena Spoltore, Marialuisa Appetecchia

**Affiliations:** 1 Oncological Endocrinology Unit, IRCCS Regina Elena National Cancer Institute, Via Elio Chianesi 53, 00144 Rome, Italy; marilda.mormando@ifo.it (M.M.); rosa.lauretta@ifo.it (R.L.); giulia.puliani@ifo.it (G.P.); marta.bianchini@ifo.it (M.B.); 2 Section of Medical Pathophysiology, Food Science and Endocrinology, Department of Experimental Medicine, Sapienza University of Roma, 00185 Rome, Italy; mariaelena.spoltore@uniroma1.it

**Keywords:** medullary thyroid cancer, cabozantinib, vandetanib, selpercatinib, adverse events, lung cavitations

## Abstract

**Background**: The current possible treatments of advanced medullary carcinoma (MTC) include different drugs belonging to the class of tyrosine kinase inhibitors (TKIs): vandetanib, cabozantinb, and selpercatinib. Although the effects of these TKIs have been well described in clinical trials, the real-practice evidence of the effectiveness and safety of these treatment is scant. This real-world case series aims to describe a niche of patients with advanced MTC treated with more than one TKI by focusing on treatment responses and any reported adverse events (AEs) and to provide additional insight on the individualized approach to the management of metastatic MTC. **Methods**: Five patients with a diagnosis of metastastic MTC, treated with at least two different molecules of TKIs, were retrospectively selected. **Results**: Three patients obtained a partial response (one with cabozantinb, one with selpercatinib, and one with vandetanib), and two patients obtained disease stability (both of them treated with all three TKIs, the first two lines discontinued for AEs). The AE profile agreed with the known clinical trials AEs except for non-neoplastic ascites related to selpercatinib and lung cavitations of non-neoplastic tissue related to cabozantinb. The latter was an AE never described so far in patients receiving TKIs. **Conclusions**: The best management of MTC relies on an individualized approach, keeping in mind and dealing with the potential toxicity in order to minimize the treatment withdrawal.

## 1. Introduction

**Figure 1:**
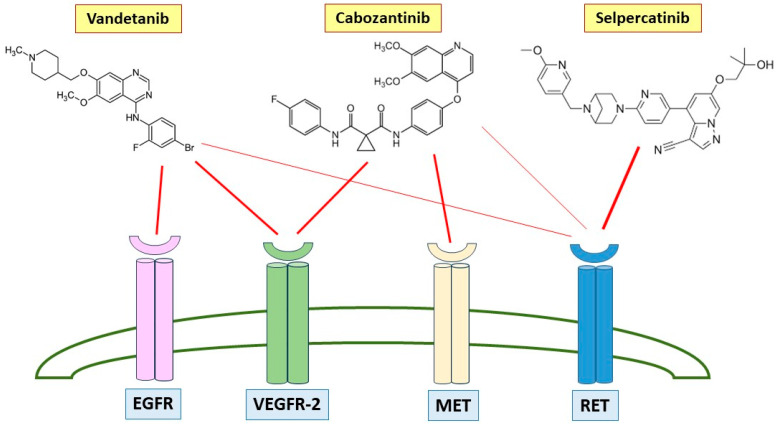
Vandetanib and cabozantinib inhibit different tyrosine kinase receptors. Vandetanib blocks EGFR (Epidermal Growth Factor Receptor), VEGFR-2 (Vascular Endothelial Growth Factor Receptor 2), and RET (Rearranged During Transfection). Cabozantinib acts on VEGFR-2, RET, and MET (Mitogen-Activated Protein Kinase). Selpercatinib is a selective RET inhibitor with higher affinity for RET compared with vandetanib and cabozantinib (the thickness of the red arrows is proportional to the affinity for the receptor).

Medullary thyroid carcinoma (MTC) is a rare neuroendocrine tumor and represents 3–4% of all thyroid cancer types. It is derived from the parafollicular cells of thyroid (C cells) producing calcitonin (CT); therefore, it is biologically distinct from more common differentiated thyroid cancer. MTC can appear as a sporadic (75%) or as an inherited tumor (25%) due to a germ-line mutation of the proto-oncogene rearranged during transfection (*RET*) coding for a receptor tyrosin kinase [1]. Its most common presentation is a solitary thyroid nodule without any symptoms. The first-line therapy and the only curative option for MTC is thyroidectomy associated with cervical lymph node dissection [2]. Different options are available in patients with recurrent MTC and distant metastases, but there is no agreement on which is the best therapeutic approach and on the right timing of starting treatment, because many patients with metastatic disease have indolent disease courses for a long time. Therefore, treatment management in these patients depends on the clinical conditions, presence of the symptoms, disease location, volume or burden, location of the metastatic lesion, and possibility of disease progression. Recurrent or metastatic MTC can be treated with surgical resection, external beam radiation therapy (EBRT), and directed local therapies when disease is localized. In the case of systemic disease, with a high disease burden, the first-line options are either vandetanib or cabozantinb, both belonging to the class of TKIs. A new target drug, selpercatinib, has been recently introduced, and the good results of the clinical trial will have an impact on the treatment scheme [3,4,5]. Figure 1 shows the molecular structures of vandetanib, cabozantinib, and selpercatinib and the receptors to which they bind, thereby inhibiting their respective intracellular pathways. The safety profile of the available TKIs should be considered in the treatment strategy, especially for patients treated with different lines of treatment. In this case series we analyzed the outcomes and side effects of different treatments for advanced MTC in patients treated with multiple lines, with the aim of providing useful information for increasingly personalized and patient-tailored medicine.

## 2. Patients and Results

We selected, from our cohort of patients with MTC, five patients who had been treated with at least two different molecules of TKIs, excluding patients who had received only one line of therapy. The order of treatment with the different lines of drugs was not equal for all patients, according to the different start times of systemic therapy for each patient and related also to the different availability of these therapies over time. For each patient the clinical case description was reported, underlining all the AEs related to each therapy, as well as the efficacy in term of disease response. The study was conducted following the ethical principles of the revised version of the Declaration of Helsinki. All patients provided informed consent to the treatment and publication of clinical data.

Below, we describe the cases of the five patients and report in Table 1 the therapies performed along with their respective AEs, duration, and reasons for therapy discontinuation.

### 2.1. Case 1: Optimal Safety Profile of Vandetanib in Patient Developing Severe Adverse Events with Cabozantinib and Selpercatinib

In 2001, a 36-year-old man underwent total thyroidectomy with a diagnosis of MTC. Afterward, seven surgical revisions were performed for the involvement of the laterocervical lymph nodes. The 6-[18F]-fluoro-L-dopa (F-DOPA) PET imaging showed the recurrence of disease in different bone segments, for which the patient underwent therapy with denosumab 120 mg monthly from June 2017 to May 2019. In July 2018, due to disease progression to the liver, chemotherapy with vandetanib at a dosage of 300 mg daily was started. The treatment was well tolerated, with the exception of a rise in blood pressure leading to the modification of antihypertensive therapy. The patient also developed transient primary hypoadrenalism, treated with cortisone acetate from November 2020 to May 2022. Vandetanib therapy determined a stable disease and led to a reduction in the serum CT. The patient was treated with vandetanib until May 2022 when, due to unavailability of the drug, he was switched to cabozantinib therapy 140 mg per day. A few days after starting the new therapy, the patient experienced severe hypocalcemia, diarrhea, and an acute rise in blood pressure, requiring hospitalization. Hypocalcemia occurred in the context of pre-existing hypoparathyroidism, already being treated with calcium and calcitriol. After increasing calcium carbonate therapy and despite normalized serum calcium levels, the inappetence and the weight loss persisted; then cabozantinib was first reduced and subsequently discontinued. The *RET* mutation in the surgical sample revealed the C618S mutation. In February 2023, CT values were increasing, and also taking into account the *RET* mutation, we switched to selpercatinib therapy at the dose of 160 mg twice daily. After only a few days, the patient experienced the onset of pronounced edemas mainly in the lower limbs and face; therefore, after 10 days, the dose was reduced to 120 mg twice a day. The blood tests showed a mild increase in the creatinine value (1.22 mg/dL) with a GFR of 64 mL/min/1.73 m^2^ and a calcium value of 9.4 mg/dL but no protein in 24 h urine. Due to worsening of the declivous edemas, a new blood assessment was performed showing a creatinine value of 1.61 mg/dL with an estimated glomerular filtration rate (eGFR) of 47 mL/min/1.73 m^2^. The hepatic and thyroid function, blood glucose, complete blood count, and urine test were within normal limits, and albumin was slightly reduced to 3.2 g/dL (normal value 3.4–4.6) with increased calcium levels (11.3 mg/dL). A color doppler ultrasound of the lower limbs showed bilateral superficial venous insufficiency, without phlebo-thrombosis, while the echocardiogram was within limits. Therefore, selpercatinib therapy was discontinued in March 2023. Since vandetanib was available again, it was decided to switch the patient back to this therapy as it was the one best tolerated by the patient. Later follow-ups showed fair tolerance to vandetanib. Creatinine values improved with the reduced vandetanib dose of 200 mg, assessing at 1.04 mg/dL with eGFR of 78 mL/min/1.73 m^2^ at the latest follow-up visit.

### 2.2. Case 2: Partial Response to Cabozantinib with an Unusual Side Effect: Cavitation in Normal Lung

**Figure 2:**
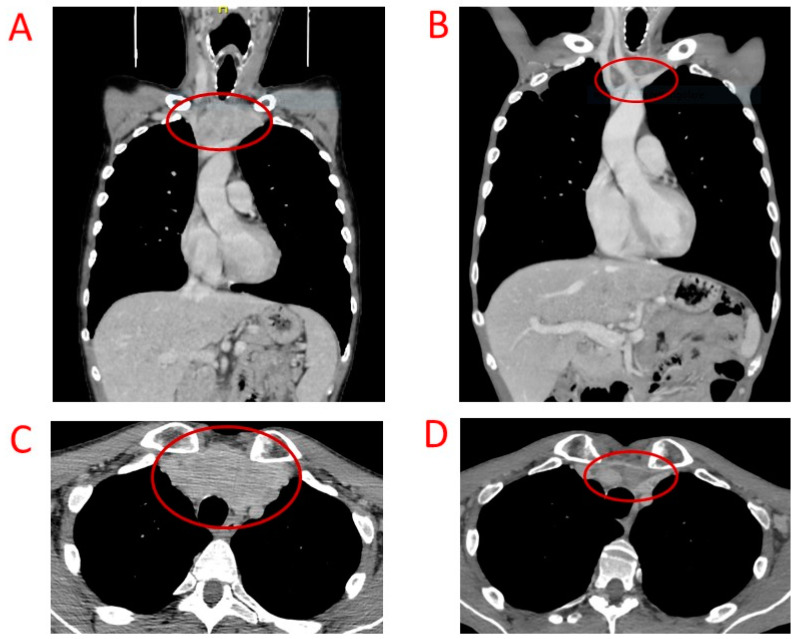
Coronal and axial scans (**A**,**C**) show the mediastinal metastatic lymphadenopathies in patient 2; in (**B**) (coronal scan) and (**D**) (axial scan), a significant shrinkage of the mass is clearly visible 6 months after cabozantinib therapy. The red circles indicate the tumor mass and its reduction.

**Figure 3:**
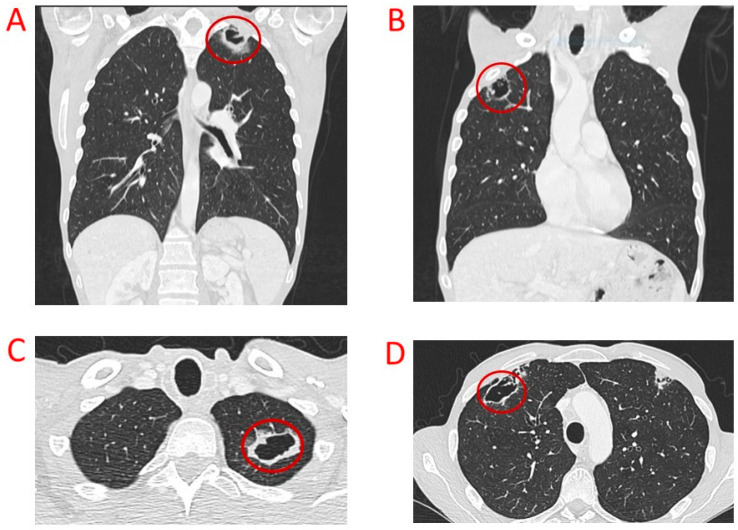
Bilateral apical lung cavitations indicated by the red circles in the normal lung parenchyma, free of metastatic lesions, were evident after 6 months of therapy with cabozantinib, as shown in the coronal (**A**,**B**) and axial (**C**,**D**) scans of patient 2.

A 45-year-old patient in March 2019 underwent thyroidectomy and lymphadenectomy for a multifocal MTC with neoplastic diffusion at cervical nodes and paratracheal fibrous and muscle tissue (pT4aN1b). Pre-surgical CT was 4953 pg/mL, and carcino-embryonic antigen (CEA) was 69 ng/mL (normal value < 5 ng/mL). After surgery, CT and CEA levels persisted as elevated with a severe paraneoplastic syndrome characterized by diarrhea, for which therapy with somatostatin analogues was started without benefit. The computed tomography (TC) scan showed a metastatic high-burden disease localized in the neck and mediastinum with a greatest lymphadenopathy of 4.5 cm located in the jugular region. The germinal mutational assessment of the *RET* oncogene showed a wild-type condition. Because of the patient’s refusal of another surgery, a first-line therapy with vandetanib was started in September 2019 with a stable disease until February 2021. Vandetanib therapy at the full dose of 300 mg daily was well tolerated without significant side effects, but paraneoplastic severe diarrhea persisted. The scheduled staging with a TC scan showed the dimensional increase in the mediastinal lymphadenopathies; then the patient was initiated to second-line therapy with cabozantinb 140 mg daily. CT levels decreased in five months from 18,943 pg/mL to 2864 pg/mL, and CEA levels decreased from 221 ng/mL to 86 ng/mL. The cabozantinb dose was reduced to 100 mg/daily due to increased hematocrit and hemoglobin levels. The patient experienced a progressive worsening of inappetence with a subsequent further weight loss (from 58 kg to 50 kg). The TC scan performed 6 months after starting cabozantinb showed a mediastinal lymph node shrinkage with a partial response (PR) (Figure 2) but a concomitant appearance of multiple bilateral lung cavitations, resulting as negative at bronchowashing for bacteria, mycobacteria, and viruses (Figure 3). However, the patient was treated with antibiotic therapy, and the TC scan imaging 4 months later showed a reduction in lung cavitations and a further shrinkage of all the mediastinal adenopathies with a necrotic reaction. Cabozantinib therapy was continued until April 2022 because the patient was unable to perform scheduled visits at our institution for progressive worsening of performance status complicated by SARS-CoV2 infection. At the same time, the molecular analysis of thyroid tissue excluded oncogene *RET* somatic mutation; hence, a switch therapy with selpercatinib had to be excluded. The last whole-body TC scan performed in September 2022 showed a stable mediastinic disease and a dimensional increase in lung cavitations characterized by thickened and irregular walls. The patient’s clinical condition progressively worsened due to persistent anorexia and recurrent lung infections; then palliative care was started up to the exitus 6 months later.

### 2.3. Case 3: High Efficacy and Safety of Selpercatinib in an Older Adult Female Patient

In June 2022, a 76-year-old female patient came to our clinic for increases in CT and CEA values (CT > 2000 pg/mL and CEA 939 ng/mL). She underwent total thyroidectomy and central lymphadenectomy in 2014 with a post-surgical diagnosis of MTC (pT2pN0), but she did not have any subsequent clinical, laboratory, and instrumental follow-up for 8 years. The F-DOPA PET showed a huge buildup of radiopharmaceuticals in both lung fields, the liver with almost complete replacement of normal parenchyma, the abdominal lymph nodes, and also the left breast. The breast biopsy showed the simultaneous presence of an infiltrating ductal breast carcinoma; therefore, a differential diagnosis on the nature of the metastatic lesions was necessary. The hepatic biopsy confirmed a localization of MTC. Breast surgery was excluded, and a therapy with aromatase inhibitors was started; in the meantime, due to the commercial unavailability of Vandetanib, in December 2022, treatment with cabozantinib was started at a reduced dose of 60 mg daily, considering both the patient’s age and the liver failure. After 20 days, proteinuria increased to more than 3 g/24 h; then treatment was discontinued. Nevertheless, proteinuria persisted for three months and in March 2023 decreased to 400 mg/24 h; the biomolecular analysis of the surgical thyroid sample showed a mutation of the *RET* oncogene (M918T) making the patient eligible for selpercatinib, a RET-selective tyrosine kinase inhibitor (TKI). Considering the patient’s age and the liver failure, selpercatinib was started in March 2023 at a reduced dose of 120 mg twice daily. In the meantime, the patient’s clinical conditions were worsening, with the presence of liver enlargement and abdominal swelling; the abdominal TC confirmed abundant peritoneal effusion for which an evacuative paracentesis was necessary, but the cytology examination excluded the presence of neoplastic cells in peritoneal fluid. Due to the increased blood creatinine levels (grade 2 of “Common Terminology Criteria for Adverse Events”, CTCAE) and QT prolongation, selpercatinib therapy was discontinued in June 2023 and restarted one month later at a reduced dose of 80 mg twice daily. Calcitonin and CEA levels progressively reduced over 11 months of selpercatinib therapy (CT from >7000 to 100 pg/mL and CEA from about 2700 to 100 ng/mL), the ascitic effusion was slightly reduced, and the CT scan in October 2023 showed a PR at the lung and abdominal lymph nodes.

### 2.4. Case 4: Non-Neoplastic Ascites During Selpercatinib Therapy

**Figure 4:**
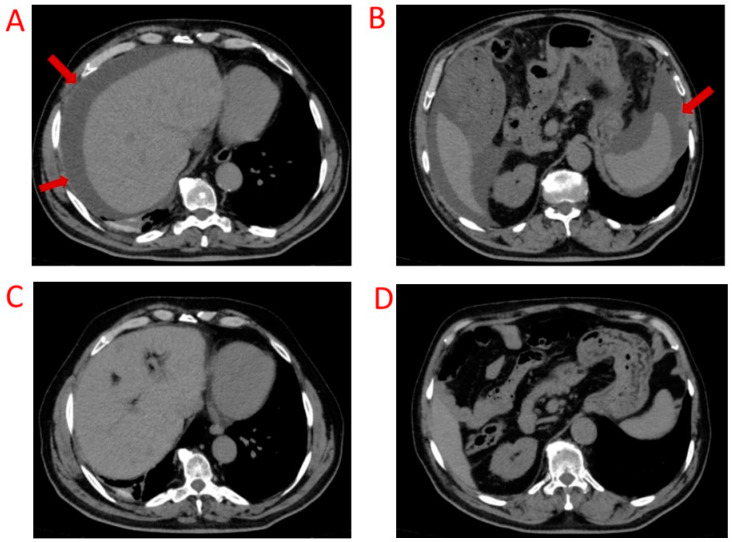
Axial scans of ascitic effusion indicated by red arrows during selpercatinib therapy (peri-hepatic in (**A**) and peri-splenic in (**B**)) and its complete disappearance 6 months after selpercatinib discontinuation (**C**,**D**) in patient 4.

In July 2019, a 71-year-old patient with suspicious MTC with cervical lymph nodes and mediastinal and pulmonary metastasis underwent total thyroidectomy with surgical removal of neck and mediastinal lesions and an atypical left lung resection. The histological examination confirmed a diagnosis of MTC (pT2 (m)pN1b), while the pulmonary nodule was found to be an amartocondroma. The surgery was complicated by a non-neoplastic pleural effusion for which the patient was treated with drainage and pleural surface. The genetic analysis of the germinal *RET* mutation was negative. CT levels never normalized, and 18 months after surgery, they reached about 50 pg/mL, while CEA was normal. The TC scan, performed in September 2020, showed mediastinal lymphadenopathies and suspected liver lesions. The specific F-DOPA PET, in December 2020, detected an increased radionuclide uptake of the mediastinal lymphadenopathies. The mutational analysis of the thyroid specimen found the M918T *RET* proto-oncogene somatic mutation. Therefore, the patient was enrolled in a phase 3, randomized trial (J2G-MC-JZJB) involving *RET*-mutant patients affected by metastatic MTC TKI-naive, comparing selpercatinib, as the first-line therapy, with cabozantinb or vandetanib. Our patient was randomized to cabozantinib therapy at a daily dose of 140 mg. The adverse events that occurred during cabozontinib therapy were measured with CTCAE and were as follows: hypertension (grade 2), dry skin (grade 1), proteinuria (grade 1), weight loss (grade 2), nausea (grade 1–2), vomiting (grade 1), hypocalcemia, and hypothyroidism. Before starting cabozantinib, CT levels were 13.7 pg/mL, and CEA was 75 ng/mL, but about one year later, in March 2022, because of disease progression of the liver and mediastinal target lesions, cabozantinib was discontinued, although CT was reduced to 1.9 pg/mL and CEA to 49 ng/mL. The patient started selpercatinib treatment at a dose of 160 mg twice daily, achieving a good recovery of body weight and complete resolution of nausea and vomiting. Unfortunately, after 9 months of therapy, in December 2022, the patient developed ascites for which a cytological investigation was performed and excluded the presence of neoplastic cells in peritoneal cavity. Hypertension and dry skin persisted during selpercatinib treatment. After 18 months the TC scan showed a further progression of the liver lesions, which was the reason why, in October 2023, the patient started a third-line TKI therapy with vandetanib at a daily dose of 300 mg. After 3 months of therapy, this treatment was well tolerated by the patient, without significant adverse events. After six months, the abdominal ultrasound and TC examination documented the complete disappearance of the ascitic effusion and PR on liver metastasis (Figure 4).

### 2.5. Case 5: The Treatment of MTC with Bone Metastasis

In a 51-year-old female patient, MTC appeared with multiple metastatic lesions at the spine, skull, and pelvic bone. The bone biopsy confirmed the thyroid origin of the neoplastic cells; then the patient, in February 2022, underwent total thyroidectomy and jugular and left laterocervical lymphadenectomy. The histological examination confirmed a 5 cm MTC and 6 out of 15 metastatic lymph nodes, with extracapsular extension (pT3N1b). Two months after surgery, the patient started systemic therapy with vandetanib at a dose of 300 mg daily with a satisfying tolerance, but only one month later, due to vandetanib’s commercial unavailability, the patient switched to cabozantinib starting at a reduced dose of 100 mg daily due to transaminases elevation caused by the strong painkiller therapy that the patient was taking for bone pain. During the 9 months of cabozantinib therapy, the dose was further reduced to 80 mg per day, and some periods of suspension were required due to the debilitating lack of appetite and weight loss. The patient lost 15 kg in 9 months. The mutational analysis of the thyroid specimen found the M918T *RET* proto-oncogene somatic mutation, although germinal *RET* mutation was negative; then selpercatinib therapy in March 2023 was initiated at a dose, by weight, of 120 mg twice daily. The patient experienced only lower limb edemas, and the dose was further reduced to 80 mg twice daily, with an excellent tolerance profile. At the same time, in May 2023, after solving dental problems, the patient started therapy with denosumab for bone metastases at a scheduled dose of 120 mg monthly. The patient gradually recovered the lost 15 kg, and to date, she is in good general condition with a dose of selpercatinib of 120 mg twice daily. The TC scans over time showed a stable disease, but CT and CEA levels reduced only with selpercatinib therapy. Before therapies, CT blood levels were 101 pg/mL (<10), while CEA was 850 ng/mL (<5). During cabozantinib therapy, CT dropped to 47 pg/mL, but CEA was unchanged; during selpercatinib therapy instead, CT normalized to 10 pg/mL, and CEA dropped to 357 ng/mL.

## 3. Discussion

MTC is one of the thyroid tumors that most frequently may present with distant metastases or that undergo progression after the initial surgical treatment. The management of metastatic MTC is challenging, especially until a few years ago, when cytotoxic chemotherapy was the only therapeutic option and had very poor outcomes [5]. Promising results in the treatment of advanced and progressive MTC were obtained 10 years ago with the development of the first multikinase inhibitors, targeting different tyrosin kinase receptors. Vandetanib and cabozantinib are the first-line therapeutic options approved for patients with advanced and progressive MTC [3,5,6], but two new targeted drugs, selpercatinib and pralsetinib, have been recently introduced, following promising results in clinical trials. The first TKI approved by the Food and Drug Administration (FDA) on April 2011 for the treatment of advanced and progressive and/or symptomatic MTC was vandetanib, which blocks signaling of epidermal growth factor receptor (EGFR), of vascular endothelial growth factor receptor-2 (VEGFR2), and of rearranged during transfection (RET). Meanwhile, cabozantinib blocks the hepatocyte growth factor receptor (MET), RET, and VEGFR2. The main activity of both drugs is versus VEGF receptors (particularly VEGFR2), while the affinity for RET is lower [1,7,8]. Both vandetanib and cabozantinib are shown to increase progression-free survival (PFS) in patients with symptomatic and progressive MTC [9,10]. Regarding the overall survival (OS), the best results appear to be related to the use of cabozantinib in patients with *RET* M918T-positive MTC (44.3 months vs. 18.9 months in those treated with placebo) [11]. More recently, highly selective RET kinase inhibitors, including selpercatinib and pralsetinib, have been approved in oncology, but the second drug has not yet been approved in Italy. Selpercatinib has been approved for adult patients with metastatic RET fusion–positive NSCLC and adult and pediatric patients ≥ 12 years of age with advanced or metastatic *RET*-mutant MTC or metastatic *RET* fusion–positive thyroid cancer who require systemic therapy and who are radioactive iodine refractory. In the registration studies (phase I–II trial, LIBRETTO-001 trial), at the dose of 160 mg twice daily, selpercatinib showed efficacy in MTC with a 1-year PFS of 82% in patients with *RET* mutant MTC who had previously been treated with vandetanib, cabozantinib, or both and of 92% in patients with *RET*-mutant MTC without previous treatment [12]. Selpercatinib, approved by the FDA in 2020 and in 2021 by the European Medicines Agency (EMA), has also been approved by the Italian Pharmaceutical Agency (AIFA) in August 2022, after the results of the phase III randomized trial (Libretto-531) comparing selpercatinib as a first-line therapy with cabozantinib or vandetanib (control group) in patients with progressive MTC. The study showed a PFS at 12 months of 86.8% (95% CI, 79.8–91.6) in the selpercatinib group and 65.7% (95% CI, 51.9–76.4) in the control group; the overall response rate (ORR) was 69.4% (95% CI, 62.4–75.8) in the selpercatinib group and 38.8% (95% CI, 29.1–49.2) in the control group [4]. Although vandetanib and cabozantinib have been associated with increased AEs due to their wide antiangiogenetic activity with a frequent dose reduction or possible discontinuation, to date, there is still no clear evidence as to which systemic regimen should be used as the first-line treatment. The potential toxicity of all the drugs above, if severe, may lead in some cases to an early discontinuation of treatment; then, a proper management of side effects is essential to ensure the patient’s compliance over the long term and thus to see the beneficial effects of the treatment, which should be continued as long as possible. We still do not know why some patients tolerate medications better than others, even if genetic makeup appears to play a role, but it is clear that higher doses are associated with more frequent and intense side effects [13]. In ZETA trial [10], vandetanib caused an AE in 55% of the patients (24% having at least grade 3 toxicities). The most common AEs were diarrhea (56%); skin abnormalities like rash, acne, and folliculitis (~50%); nausea (30%); hypertension (30%); abdominal pain, fatigue, headaches, and decreased appetite (20–25%); and proteinuria (10%). Fourteen percent of the patients had prolongation of the QT interval, a potentially life-threatening condition because it can predispose to torsade des pointes. Due to the side effects, 35% of the patients needed dose reductions, and 12% discontinued treatment. Five patients (2%) had treatment-related lethal toxicity, but their causes of death were not clearly related to prolongation of the QT interval. Rare but serious AEs included interstitial lung disease, Stevens–Johnson syndrome, cerebrovascular accidents, congestive heart failure, and reversible posterior leukoencephalopathy. The AEs of cabozantinib were reported in the EXAM trial [11]. The most common were diarrhea (63%), hypothyroidism, hypocalcemia, stomatitis, hand–foot syndrome, weight loss, nausea, lack of appetite, fatigue (40–50%), and hypertension (30%). Of the patients, 79% percent needed dose reductions, and 16% discontinued treatment. Of the patients, 69% had grade 3 or 4 AEs, and 6% experienced a fatal AE. Rare but serious AEs included gastrointestinal perforation, fistula formation, bleeding, thromboembolic phenomena, osteonecrosis of the jaw, poor wound healing, and reversible posterior leukoencephalopathy. The LIBRETTO-001 trial [14] described the efficacy and safety of selpercatinib in 702 patients, 143 of them *RET*-mutant, 55 previously treated with cabozantinib or vandetanib, and 88 patients naive. The most common severe AEs were hypertension (grades 1–4: 35% any grade, 18% grade 3–4), increased alanine aminotransferase (ALT) (45% any grade, 9% grades 3–4), increased aspartate aminotransferase (AST) (51% any grade, 8% grade 3–4), increased creatinine levels (37% any grade, 1% grade 3–4), and edemas (33% any grade, 0.3% grade 3–4). The LIBRETTO 531 comparing selpercatinib with vandetanib or cabozantinb as a first-line therapy confirmed the same AEs, including among the most common (>20%) also diarrhea (26%), dry mouth (32%), and fatigue (19%). In the LIBRETTO 531 trial, the AEs led to a dose reduction in 38.9% of the patients in the selpercatinib group, as compared with 77.3% in the control group, and to treatment discontinuation in 4.7% and 26.8%, respectively.

Due to the relatively recent introduction of these drugs, no consistent real-world data are available. A real-world multicenter retrospective experience conducted on 12 metastatic patients with locally advanced MTC treated with vandetanib demonstrated a PR in five patients (42%) and stable disease for ≥24 weeks in an additional five patients (83%) with a median PFS of 25.9 months [15]. Only one larger real-world study involving 48 progressive MTC patient treated with vandetanib and/or cabozantinib [16] confirmed their efficacy in this patient setting. A total of 47 patients (98%) were treated with vandetanib and 23 (48%) with cabozantinib (first- or second-line treatment), and a PR was observed in 26% of patients treated with vandetanib and 22% with cabozantinib. The median PFSs for vandetanib and cabozantinib were 17 months and 4 months, respectively. Both in patients treated with vandetanib and in those treated with cabozantinib, the PFS was significantly longer in patients experiencing AEs and in patients aged ≤60 years. In the vandetanib group the PFS was longer in the absence of bone metastases, but in this patient setting, vandetanib was more effective than cabozantinib (PR in 28% vs. none). The poorer prognosis of cabozantinib-treated patients was likely due to its use as a second-line treatment after vandetanib failure and in rapidly progressive diseases.

In our experience, described in this case series, we are aware that this is a very small cohort, but given the rarity of the disease, we wanted to describe our monocentric experience with recently introduced drugs for which there are still limited real-life data on efficacy and all potential side effects. Three out of five patients were treated, in a different order, with all of the three drugs currently available in Europe for metastatic MTC (vandetanib, cabozantinb, and selpercatinib) and two of them with only two drugs (vandetanib/cabozantinib and cabozantinib/selpercatinib). Three patients obtained a PR, one of these with cabozantinb (patient 2), one with selpercatinib (patient 3), and one with vandetanib (patient 4); all these responses were evident after 6 months of therapy. Disease progression occurred in patient 2 with the first-line therapy with vandetanib (PFS of 17 months) and in patient 4 with cabozantinib and selpercatinib (with PFS of 12 months and 18 months, respectively). Disease stability was evident in two patients, both of them with bone metastases (patients 1 and 5). A *RET* somatic mutation was observed in four of five patients (M918T mutation in three cases and C618S in one case). A disease PR was achieved in the wild-type *RET* patient with cabozantinb and in the two patients with M918T mutations with selpercatinib and vandetanib. Although responses to therapy were seen in a relatively short time (6 months), in some cases it was not possible to evaluate the real efficacy of selpercatinib and cabozantinib, due to early discontinuation for the AEs that were mostly in line with those reported in clinical trials and all reversible. In three of the patients, the first-line therapy with vandetanib was interrupted due to the drug’s unavailability, without significant side effects or disease progression. It is worth mentioning that patient 5 was only on vandetanib for one month, so its efficacy could not be evaluated. For the second-line therapy, three out of five patients were treated with cabozantinib, and in all cases, treatment was discontinued due to side effects. Two patients received selpercatinib as a second-line therapy; in one case, treatment continued with a partial response, while in the other, it was stopped due to disease progression (Table 1).

From our experience we must point out two AEs not described in clinical trials probably due to the low prevalence: the lung cavitations on non-neoplastic tissue developed during cabozantinib therapy and the non-neoplastic ascites associated with selpercatinib therapy.

Regarding the lung cavitations, the antiangiogenetic agents have been associated with the development of cavitations of lung neoplastic lesions that can be explained with the necrosis treatment-related, cyst formation or desquamation/liquefaction of tumor cells within the lesion [17]. Several authors reported tumoral cavitations in advanced lung cancer during antiangiogenetic therapy [18,19]. To date, pulmonary cavitations are an unexpected AE in patients with metastatic thyroid cancer, but a recent retrospective cohort study conducted on 83 patients affected by thyroid cancer and lung metastases treated with multi-targeted antiangiogenic TKIs described 10 cases of lung cavitations (12%), two of them complicated with pneumothorax although without effects on survival [20]. The impact of tumor cavitation on survival in lung cancer patients treated with antiangiogenetic drugs remains controversial: studies conducted on different lung cancer histotypes have been associated with a better or worse PFS [21,22]. To the best of our knowledge, we described for the first time the case of lung cavitations arising on healthy tissue in a patient treated with a TKI drug, specifically with cabozantinib. The pathogenic mechanism underlying this event remains difficult to explain: we could consider the possible cumulative anti-angiogenic mechanism of the two drugs since the patient had been treated with vandetanib before cabozantinib, or we could consider the possibility of unknown genetic predisposing factors. Certainly, our findings will need to be confirmed by further studies. It is essential to take into account this AE since it compromised the patient’s outcome and predisposed him to recurrent pneumonia and infections, which did not allow the continuation of oncological treatment. Unfortunately, the pulmonary cavitations were not responsive to antibiotic treatments, so this AE contributed, together with anorexia and severe weight loss, to the cabozantinb discontinuation, despite a satisfactory response on the other target lesions.

Currently, most of the information regarding the AEs associated with selpercatinib is derived from clinical trials, but long-term or rare AEs could be encountered from the clinical practice. The occurrence of effusions during selpercatinib treatment, although not reported in LIBRETTO-001 trials [14], has been recently described in a study using the FDA Adverse Event Reporting System Database [23]. In this study, three new significant AEs were described in a total of 464 reports and 1007 selpercatinib-related AEs: dysphagia, pericardial effusion, and hemiparesis. A retrospective multicenter study reported spontaneous chylous effusions associated with selpercatinib with an incidence of 7%, more than other TKIs [24]. Furthermore, a retrospective study evaluating MCT patients enrolled in the LIBRETTO-201 clinical trial (ClinicalTrial.gov Identifier NCT03906331) described the presence of effusions in 8/10 MTC patients treated with selpercatinib. Five of them had no effusions before starting selpercatinib, and three had some effusions already present before selpercatinib but developed new effusions in other sites (pleural, pericardial, abdominal, or pelvic) and/or worsened those that were already present. Fluid aspiration, performed in symptomatic and/or moderate/severe cases, demonstrated the chylous nature of the effusions and the absence of malignant cells. The dose reduction has proven effective on related symptoms [25]. Several etiologies of chylothorax or chylous ascites have been described, but in that cohort [25], congenital, traumatic, and obstructive causes have been excluded. Although the mechanism underlying chylous effusions remains unclear, given the potential role of the RET protein in the migration of lymphatic endothelial cells [26], a possible hypothesis is that RET inhibition could alter the integrity of lymphatic endothelial cells, leading to increased lymphatic endothelial permeability and ultimately resulting in chylous effusions. Also, in our experience the ascitic effusion was considered a selpercatinib-related AE in patient 4 but not certainly in patient 3, since the presence of ascites was found simultaneously with the start of selpercatinib therapy, and we can suppose a possible role of the previous cabozantinib therapy. In patient 4, the cytological analysis excluded the presence of malignant cells, although it did not analyze the nature of the effusion, and this AE was shown to be reversible with discontinuation of the drug.

In our clinical experience, vandetanib demonstrated an optimal efficacy profile in terms of disease stabilization and long-term tolerability, with a treatment duration of up to 48 months in one patient and mostly at the maximum dose of 300 mg daily without a dose reduction requirement. Undoubtedly, the longer-lasting experience on vandetanib may have led to better management of AEs, compared with cabozantinib and selpercatinib, which, however, demonstrated good efficacy in terms of response to treatment, and consequently, they may be more effective in patients with rapidly progressing MTC. Due to the little consensus available on how to select a specific treatment as the first line [5], actually, each of the three drugs can be chosen. Mutation analyses are yet not useful to indicate which patients are most likely to benefit from targeted treatment, although current evidence favors vandetanib and selpercatinib in cases of *RET* oncogene mutation. The selection of the first-line systemic therapy for MTC must be individualized, considering patients’ characteristics, such as their performance status, age, physical examination, comorbidities, other treatment, social aspects, disease progression, tumor features, and medication availability. Advanced MTC with bone metastases remains one of the most urgent challenges since the objective response in these patients is very poor, and studies to date have not demonstrated the superiority of any treatment over another. In our patients with bone metastases, stable disease was observed during vandetanib, cabozantinib, and selpercatinib, but a significant reduction in CT and CEA levels was obtained only with selpercatinib. Our limited case series comparing different treatment regimens does not allow us to determine the most effective therapy for patients with metastatic MTC. What stands out from our experience is the importance of preventing severe side effects that could lead to therapy discontinuation. Specifically, with cabozantinib, it is crucial to closely monitor proteinuria, electrolyte imbalances (especially hypocalcemia), and weight loss. Therefore, nutritional support and adequate supplementation of calcium and magnesium are essential, particularly in patients with post-surgical hypoparathyroidism.

## 4. Conclusions

TKIs are valuable treatments in patients with non-resectable symptomatic, rapidly progressive MTC because they can extend PFS and provide long-term disease stabilization. However, complete responses are uncommon, and TKIs have many significant adverse effects, which can lead to poor quality of life. The best option for the management of MTC must be an individualized approach, keeping in mind the patient’s characteristics, the potential toxicity, and the effects of the treatment on their quality of life in order to minimize the dose reduction or treatment withdrawal. Specifically, cabozantinib should be used with caution in patients with post-surgical hypoparathyroidism and malabsorption issues. In the case of selpercatinib, special attention should be given to patients with hepatic metastases or cardiovascular diseases due to the risk of ascites and pericardial effusion. Lung cavitations in the absence of pulmonary metastases should be considered as a possible adverse event with cabozantinib therapy.

## Figures and Tables

**Table 1:** Summary of the therapy sequences administered to each patient, including their associated adverse events, duration, and reasons for discontinuation.

	I Line Therapy (Duration and Efficacy)	Adverse Events	Reason for Stop	II Line Therapy (Duration and Efficacy)	Adverse Events	Reason for Stop	III Line Therapy (Duration and Efficacy)	Adverse Events	Current Therapy	Last Follow up/Death
**Patient 1**	Vandetanib (46 months; SD)	Hypertension (G2) Transient hypoadrenalism (G2)	Drug unavailable	Cabozantinib (9 months; SD)	Hypocalcemia (G3) Diarrhea (G2) Weight loss (G1) Inappetence (G2)	Hypocalcemia (G3)	Selpercatinib (2 months; NA)	Face and limbs edema (G1) Renal failure (G2)	Vandetanib	SD
**Patient 2**	Vandetanib (18 months; SD)	Hemoglobin increased (G1)	Drug unavailable	Cabozantinib (14 months; PR)	Weight loss (G2) Hemoglobin increased (G2)	Weight loss (G2) Pneumonia	−	−	−	Death
**Patient 3**	Cabozantinib (3 months; NA)	Proteinuria (G2)	Proteinuria (G2)	Selpercatinib (21 months- ongoing; PR)	Renal failure (G2) QTc prolongation (G2)	−	−	−	Selpercatinib	PR
**Patient 4**	Cabozantinib (18 months; PD)	Hypertension (G2) Dry skin (G1) Proteinuria (G1) Weight loss (G2)	PD	Selpercatinib (18 months; PD)	Ascites (G2) Hypertension (G2) Dry skin (G1)	PD	Vandetanib (14 months- ongoing; PR)	None	Vandetanib	PR
**Patient 5**	Vandetanib (1 month; NA)	None	Drug unavailable	Cabozantinib (10 months; SD)	Weight loss (G3)	Weight loss (G3)	Selpercatinib (21 months; SD)	Lower limbs edema (G2)	Selpercatinib	SD

Abbreviations: NA, not applicable due to the short duration of therapy; SD, stable disease; PR, partial response; G1, grade 1 CTCAE; G2, grade 2 CTCTA; G3, grade 3 CTCAE.

## Data Availability

All data generated or analyzed in this case series are included in this article. Further inquiries can be directed to the corresponding author.
